# The added value of kinematic evaluation of the timed finger-to-nose test in persons post-stroke

**DOI:** 10.1186/s12984-017-0220-7

**Published:** 2017-02-10

**Authors:** Gudrun M. Johansson, Helena Grip, Mindy F. Levin, Charlotte K. Häger

**Affiliations:** 1 Department of Community Medicine and Rehabilitation; Physiotherapy, Umeå University, Building 15, SE-901 87 Umeå, Sweden; 2 School of Physical and Occupational Therapy McGill University, 3654 Promenade Sir William Osler, Montreal, Quebec H3G 1Y5 Canada

**Keywords:** Stroke, Kinematic assessment, Arm, Hand, Validity

## Abstract

**Background:**

Upper limb coordination in persons post-stroke may be estimated by the commonly used Finger-to-Nose Test (FNT), which is also part of the Fugl-Meyer Assessment. The total movement time (TMT) is used as a clinical outcome measure, while kinematic evaluation also enables an objective quantification of movement quality and motor performance. Our aims were to kinematically characterize FNT performance in persons post-stroke and controls and to investigate the construct validity of the test in persons with varying levels of impairment post-stroke.

**Methods:**

A three-dimensional motion capture system recorded body movements during performance of the FNT in 33 persons post-stroke who had mild or moderate upper limb motor impairments (Fugl-Meyer scores of 50–62 or 32–49, respectively), and 41 non-disabled controls. TMT and kinematic variables of the hand (pointing time, peak speed, time to peak speed, number of movement units, path ratio, and pointing accuracy), elbow/shoulder joints (range of motion, interjoint coordination), and scapular/trunk movement were calculated. Our analysis focused on the pointing phase (knee to nose movement of the FNT). Independent *t* or Mann-Whitney U tests and effect sizes were used to analyze group differences. Sub-group analyses based on movement time and stroke severity were performed. Within the stroke group, simple and multiple linear regression were used to identify relationships between TMT to kinematic variables.

**Results:**

The stroke group had significant slower TMT (mean difference 2.6 s, *d* = 1.33) than the control group, and six other kinematic variables showed significant group differences. At matched speeds, the stroke group had lower accuracy and excessive scapular and trunk movements compared to controls. Pointing time and elbow flexion during the pointing phase were most related to stroke severity. For the stroke group, the number of movement units during the pointing phase showed the strongest association with the TMT, and explained 60% of the TMT variance.

**Conclusions:**

The timed FNT discriminates between persons with mild and moderate upper limb impairments. However, kinematic analysis to address construct validity highlights differences in pointing movement post-stroke that are not captured in the timed FNT.

## Background

Coordination of upper limb movements is often impaired after a stroke. This study aimed to kinematically evaluate in depth upper limb coordination in persons post-stroke using a common clinical pointing test, the Finger-to-Nose test (FNT) [1]. The FNT is usually scored by the time to complete the task, while our goal was to assess the added value of a kinematic analysis of the test. According to the framework of the International Classification of Functioning, Disability, and Health [2], coordination of simple and complex voluntary movements involves performing movements in an orderly combination (Body Functions), and performing coordinated actions such as carry, move and handle objects (Activities). The concept of coordination is complex and there is still a lack of consensus around a clear definition [3]. Bernstein viewed coordination as the process of mastering the redundant degrees of freedom involved in a particular movement, and that motor redundancy was considered as a source of computational problems solved by a unique solution [4]. In this paper, the operational term of coordination is referred to as the spatiotemporal relationship between component parts [5].

In clinical practice, the FNT is an established test used to assess upper limb coordination [6]. FNT is also included in the Fugl-Meyer Assessment for the upper extremity (FMA-UE) that evaluates upper limb impairments following stroke [7]. Most commonly the person is seated and upon command moves the index fingertip back and forth between the ipsilateral knee and the tip of the nose five times, as fast and as accurately as possible. The quantitative clinical main outcome measure of the FNT is total time of performance, which is considered more reliable than the qualitative scoring of dysmetria and tremor on a ordinal rating scale [1, 8], as it is performed in the FMA-UE [7]. Compensatory movement strategies of the trunk and/or shoulder are however not taken into account. As persons post-stroke often use compensatory movement strategies to accomplish upper limb tasks, such movements are important to consider when evaluating upper limb recovery [9]. Kinematic evaluation offers more detailed characteristics of motor performance than merely the total time to perform the task, since it provides comprehensive and informative data about joint motion sequencing and timing involved in coordinated movement. To the best of our knowledge, there are no studies that have used kinematics to quantify motor performance during the FNT, as used by Fugl-Meyer et al. [10].

There are several kinematic variables that have been shown to be highly reliable and valid to characterize pointing and reaching movements in persons post-stroke [11–14]. Motor performance, targeting aspects of movement quality, is usually assessed in terms of temporal (movement time, speed, smoothness) and spatial (joint angles, target errors, compensatory trunk movements) parameters, which may be obtained from joint and body segment kinematics and calculations of end-point positioning [12, 15, 16]. Smoothness of the movement path is considered an important characteristic of well-coordinated movement [17, 18], and a relevant variable that discriminates between persons post-stroke and non-disabled controls [12, 17–20] as well as between persons with different levels of stroke severity [12, 19, 20]. Previous studies in persons post-stroke have characterized motion deficits during pointing tasks to external targets, (e.g., away from the body) with [20, 21] and without vision [19, 22, 23]. Persons with stroke have prolonged movement times, smaller movement amplitudes, more variable upper limb movements, and disrupted elbow-shoulder coordination in the affected arm compared to the non-affected arm, as well as compared to non-disabled controls [19, 20, 23]. In addition, persons with moderate-to-severe stroke use excessive trunk movements compared to persons with mild stroke when making forward pointing movements [20]. Despite lower speed, pointing movements in persons post-stroke are less precise than those of non-disabled controls and decreases in movement accuracy correlate with level of stroke severity [19, 20]. A few studies have investigated reaching-to-mouth tasks [12, 24, 25], in which upper limb movements are similar to those used for the FNT, although the tasks have different visual conditions, accuracy, and time constraints. In some of these studies, compensatory movements such as increased shoulder abduction have been reported [12, 25].

Despite increased research of upper limb movements in persons post-stroke during recent decades [26], there are few studies of pointing movements to body-related targets, and sensorimotor control is not well understood. Detailed understanding of reaching movements undertaken in prevailing clinical tests are needed in order to fully comprehend their constructs and measurement properties [27]. Therefore, it is important to investigate the construct validity of FNT as a test of coordination within persons with different functional levels post-stroke. In the present paper, we hypothesized that the kinematic variables would substantially contribute to reveal the inherent ability of the FNT to capture upper limb coordination and compensation.

Hence, we aimed to characterize the FNT performance in a group of persons post-stroke and a non-disabled control group with regard to kinematic variables of the hand (pointing time, peak speed, time to peak speed, number of movement units, path ratio, and pointing accuracy), elbow/shoulder joints (range of motion, interjoint coordination), and scapular/trunk movement. The second aim was to address two aspects of construct validity; i) to compare FNT performance between persons with varying levels of impairment post-stroke, and ii) to determine if the FNT is valid test of coordination by relating the clinical main outcome measure of the FNT, i.e., total movement time (TMT), to the kinematic variables of the pointing phase of the test.

## Methods

### Participants

Thirty-three persons with stroke (21 men, mean age 68 ± 10 years, onset 24 ± 19 months) and 41 non-disabled controls (22 men, mean age 66 ± 12 years) participated in this cross-sectional study (Table 1). The participants post-stroke were recruited from two clinics in Northern Sweden and met the following criteria: (a) adults aged 35–85 years old, (b) residual unilateral hemiparesis following an ischemic or hemorrhagic stroke, (c) at least 3 months after stroke, (d) medically stable, (e) able to voluntarily lift the hand to the nose, (f) able to understand both verbal and written information, and (g) no impairments or diseases other than stroke that influenced upper limb movements. The control group was recruited among staff and acquaintances and through an organization for retired persons. The controls had no known musculoskeletal or neurological movement problems. Participants in both groups were right-handed except for one person post-stroke and two controls. All participants signed informed consent forms and the study was approved by the Regional Ethical Review Board in Umeå, Sweden (dnr 2011-199-31 M).

**Table 1:** Participant Characteristics^a^

Characteristic	Stroke group (*n* = 33)	Control group (*n* = 41)
Sex (male/female), *n*	21/12	22/19
Age, years	68 (10)	66 (12)
Body Mass Index, kg/m^2^	27.5 (3.3)	24.8 (2.2)^b^
Grip strength, kg (aff/non-dom)	24.8 (10.7)	35.2 (9.2)^b^
Grip strength, kg (non-aff/dom)	33.8 (9.3)	36.6 (9.6)
Handedness (right/left), *n*	32/1	39/2
Time since stroke, months	24 (19)	N/A
Side of paresis (right/left), *n*	13/20	N/A
Etiology (infarct/hemorrhage), *n*	28/5	N/A
FMA UE (0–66)	52 (9)	N/A
Impaired proprioception (subscale FMA-UE), yes/no	3/30	N/A
Spasticity in the affected arm, yes/no	11/22	N/A

^a^Measurements are reported as mean (standard deviation) unless otherwise reported. *FMA UE* upper extremity part of the Fugl-Meyer Assessment (maximal score 66), *aff* affected arm (stroke), *non-dom* non-dominant arm (control), *N/A* not applicable. ^b^Significant difference

### Clinical assessment

Clinical outcomes of the participants are reported in Table 1. For the stroke group, motor impairment was assessed with the FMA-UE [7]. The 33-item scale consists of three response categories (scores 0–2) for each item, with a maximum score of 66, indicating no impairment. The stroke group scored between 32 and 64 on the FMA-UE, and were considered to have mild to moderate motor impairments [11]. In addition, impaired proprioception was assessed with a subscale of FMA-UE (scores 0–8), where a total score of 8 corresponds to no impairment. Three persons post-stroke had decreased proprioception in thumb and elbow joints (5 or 6/8 points). Muscle tone was tested in shoulder abductors, elbow flexors, wrist flexors, and finger flexors. The Modified Ashworth Scale grades resistance to passive movement of the resting muscle on a 6-point ordinal scale ranging from 0 (no increase in muscle tone) to 4 (affected part rigid in flexion or extension) [28]. Spasticity was defined as ≥ 1+ score of Modified Ashworth Scale in one or more muscles tested. Spasticity was present in eleven persons post-stroke, of whom six persons had spasticity in all flexor muscles tested.

Grip strength was measured with a digital hand dynamometer (Jamar®, US), as the mean of three trials. Grip strength was approximately 30% lower in the affected arm of participants post-stroke compared to the non-dominant arm of controls. The first author (GMJ) performed all the clinical assessments.

### Kinematic testing protocol

**Fig. 1:**
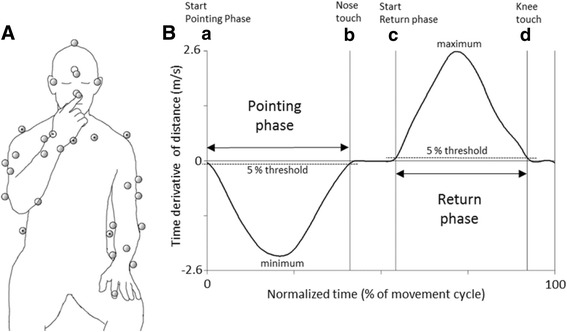
Marker set up, events and normalized phases of the Finger-to-Nose test. **A**) The unfilled marker was positioned on the dorsal side of the head, and the markers with a center dot were positioned on the trunk. Three markers (placed on nose, right and left medial epicondyles) were removed after recording a static trial for modelling purpose. Left hand shows the start position (Knee touch) and right hand shows the end position (Nose touch) for the pointing phase. **B**) Normalized phases of the Finger-to-Nose-Test based on the time derivative of distance (a negative value denotes that the finger moves towards the nose, and a positive value denotes that the finger moves from the nose). Note that the end of the pointing phase is also the event ‘Nose touch’. Likewise, the end of the return phase is when the hand reaches the knee, for the event ‘Knee Touch’. For detailed definitions of the events **a–d**, see Methods

The participants were seated in a stable height-adjustable chair (Mercado Medic REAL® 9000 PLUS) that was adjusted for each participant with their back supported but not restrained. The standardized procedure of the FNT was demonstrated by the test leader and was then imitated once by the participant to ensure comprehension of the task. Participants sat with their eyes closed and the palm of their hand on the ipsilateral knee (Fig. 1). The eyes-closed condition was verified by video recording. Participants were instructed to, on a verbal command, touch their nose with the tip of their index finger as quickly and as accurately as possible, and then return the hand to the starting position a total of five times before stopping. The stroke group performed the test with the non-affected arm first followed by the affected arm, while the control group started with the dominant arm. We compared the kinematics between the non-dominant arm of the control group and the affected arm of the stroke group, since movement kinematics of the non-dominant arm of healthy persons might be more evenly matched with those of the affected arm.

Upper body kinematics were recorded with an 8-camera 3-D motion capture system (240 Hz, Oqus®, Qualisys Gothenburg, Sweden). The 31-marker setup is presented in Fig. 1. Two video cameras (Canon Legria HV40), integrated in the motion capture system, recorded pointing movements in the sagittal and frontal planes. Data were collected in Qualisys Track Manager (QTM, version 2.6; Qualisys, Gothenburg, Sweden), and exported to Visual 3D version 5 (C-motion Inc., Germantown, MD, USA). All data were filtered at 6Hz with a Butterworth filter prior to calculations. The kinematic model used for calculations was constructed according to a Visual3D hybrid 6° of freedom model. Segments for assessing upper limb movements were the head, thorax, upper arms, forearms, and hands. The Euler sequence XYZ was used for the elbow joint while XZY was used for the shoulder joint to better define pure shoulder abduction and flexion. In both cases, +X represented flexion, +Y represented abduction and + Z represented inward rotation.

### Data analysis

Temporal and spatial variables considered relevant for a pointing task were analyzed [11, 16]. We chose to define the events based on the time derivative of the distance between the finger marker and the nose marker (see Fig. 1): a) The beginning of the *Pointing phase* was defined as the time point at which the time derivative of the distance fell below a threshold set to 5% of the *minimal* value, and remained below this threshold for at least 40 ms; b) The end of the *Pointing phase* was defined as the time point at which the time derivative of the distance exceeded a threshold set to 5% of the *minimal* value, and remained above this threshold for at least 40 ms. This event was denoted *Nose touch;* c) For the *Return phase*, the beginning was defined as the time point at which the time derivative of the distance exceeded 5% of the *maximal* value and remained above it for at least 40 ms; d) Finally, the end of the *Return phase* was consequently defined as the time point at which the time derivative of the distance fell below 5% of *maximal* value and remained below for at least 40 ms. This event was denoted *Knee touch.*

Our analysis focused on the Pointing phase that is the movement between the time when the fingertip left the knee until when it touched the nose (Nose touch). The clinical outcome (total time of performance) was obtained as *Total movement time (TMT,* s*)* of the entire test (i.e. the time from when the fingertip left the knee in the first trial and eventually returned to the knee (Knee touch) after the fifth trial). *Pointing time* (s) was the duration of the Pointing phase. *Peak speed (*mm/s*)* was defined as the maximum tangential velocity that the index finger attained during the Pointing phase, while *Time to Peak speed* (*TPS*) was expressed in seconds and as a percentage (*TPS%*) of the Pointing phase in which the Peak speed occurred. The TPS reflects the proportion of time spent between the start of the movement and Peak speed. Movement smoothness during the Pointing phase was quantified by computing the *Number of movement units (NMU)* of the index finger marker by calculating the number of local maxima in the tangential velocity curve. A cut-off value corresponding to 10% of the peak velocity was used to avoid erroneously detected movement units when no movements occurred. According to this definition, a smooth, graceful movement would have only one movement unit. Movement efficiency, or straightness, was estimated by the *Path ratio*, which is the ratio of the distance of the actual movement path and the path distance of an ideal straight line. The path ratio of the index finger marker was calculated for the Pointing phase. To analyse the spatial variability of finger position at the time of the nose touch, the *Variable error* (mm) [29] was calculated, defined as the root mean square of the distance between the index finger tip position at the event Nose touch, and the mean of all these positions, all relative to the nose. The overall measure of how successful the participant was in reaching the nose was assessed by computing the *Total variability* (mm) [29], defined as the root mean square of the distance between the index finger tip and the nose tip taken at Nose touch. End-point errors (e.g., Total variability and Variable error) correspond to movement accuracy [16]. The temporal *Interjoint coordination (IJC)* for elbow flexion and shoulder flexion was computed using cross-correlation analysis at zero time lag [13]. A correlation coefficient closer to 1.0 indicates stronger correlation and signifies that motion of the two joints is tightly coupled. In this study, the coordination between shoulder flexion and elbow flexion was of interest as concurrent flexion motions are demonstrated in persons post-stroke during reaching upwards to a target [30]. *Range of motion* (*ROM,* degrees) for each joint was defined as the difference between the maximum and minimum values of the angular joint motion curve. Excessive scapular and trunk movements were computed as the displacement of the acromion marker in the sagittal plane during the Pointing phase. *Acromion displacement* (mm) represents a global measure of sagittal plane excursion that involves scapular movements relative to the trunk as well as trunk movements. *Nose displacement* (mm), as a control for head motions during the task, was computed as the maximal displacement of the nose marker in the sagittal plane during the entire test. Positive values indicate anterior displacement while negative values denote posterior displacement.

All variables except TMT were computed as means of all five trials and then subsequently used in the statistical analysis. To address whether group differences in movement parameters were only related to slower movements in the stroke group compared to controls, we analyzed a sub-sample of two groups formed by pairing each stroke subject with a control who had comparable movement times (time difference within 0.5 s or less), which resulted in 22 pairs. Further, to test if the movement parameters were sensitive to the stroke severity, data were analysed according to two sub-groups of stroke subjects defined by FMA-UE scores ≥50/66 (Mild) and ≤49/66 (Moderate-to-severe) [11].

### Statistical analysis

Statistical analysis was performed with IBM SPSS (Statistical Packages for Social Sciences, 21.0). *T*-tests for independent samples were used for comparisons between groups. Mann-Whitney U tests were used for comparisons between two different subgroups based on a) movement time and b) stroke severity. The level of statistical significance was set to *P* < 0.05. Since the number of outcome variables of interest was large, we performed Bonferroni correction for multiple tests (0.05/48) and chose to also present this even more rigorous interpretation of the significance level (*P* ≤ 0.001). To estimate effect sizes within groups, the parametric test Glass’s *d* (mean difference/standard deviation of the controls) [31] was used, while the *r* tested effect sizes between subgroups by using the *z*-value from the nonparametric statistics [32]. Cohen’s guidelines for interpreting *d* are 0.8 = large, 0.5 = medium and 0.2 = small and for *r* 0.5 = large, 0.3 = medium, and 0.1 = small sizes [32].

For the stroke group, relationships between different variables were estimated with Spearman’s correlation coefficients and multiple linear regression. The strength of correlation was interpreted according to Munro: 0.00–0.25 = little if any, 0.26–0.49 = low, 0.50–0.69 = moderate, 0.70–0.89 = high and > 0.90 = very high correlation [33]. Analyses were conducted to ensure that there were no violations of assumptions of normality, linearity, multicollinearity and homoscedasticity. First, simple linear regression analysis was used to calculate the linear relationship between each independent variable and TMT. Then, multiple linear regression with backward deletion was used to assess how much variance in TMT could be explained by a combination of selected kinematic variables. Three independent variables of the Pointing phase with the highest strength of correlation to the TMT were initially entered in the regression model. As Peak speed and TMT both are speed related variables, Peak speed was not entered in the calculations of correlation and regression. Probability for entry in backward regression was set at 0.05 and removal at 0.10. Adjusted *R* ^*2*^ value, unstandardized coefficient (B) with their standard errors (SE) and significance values, and unique partial coefficients were reported.

## Results

**Fig. 2:**
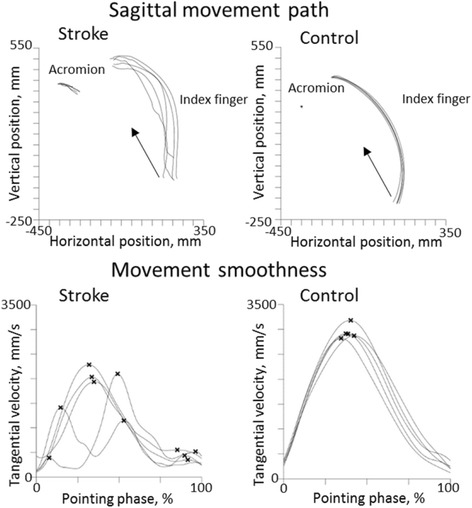
Examples of movement paths and velocity profiles from the stroke group and the control group. Movement paths from the pointing phase of the markers of the index finger and the acromion in the sagittal plane, and velocity profiles with marked movement units of one person post-stroke (*left panel*) and one control person (*right panel*). The arrow indicates the direction of the movement

The stroke group had on average a higher body mass index, and lower grip strength in the affected arm compared to the non-dominant arm of the controls (*P* ≤ 0.001 in both cases). There were no other differences in demographic characteristics between the groups (Table 1). Figure 2 shows examples of movement paths with increased variability and increased NMU for a representative participant in the subgroup with moderate stroke compared to a representative control subject.

### Motor performance during of the finger-to-nose test

The stroke group performed the FNT significantly slower than the controls (mean difference = 2.6 s; *P* ≤ 0.001, *d* = 1.33), as shown in Table 2. For the temporal variables; the stroke group had increased Pointing time (mean difference = 0.20 s, *P* ≤ 0.001, *d* = 1.48), decreased Peak speed (mean difference 550 mm/s, *P* ≤ 0.001, *d* = 1.10), and increased TPS (mean difference = 0.05 s, *P* ≤ 0.001, *d* = 0.99), compared to the control group. For the spatial variables; the stroke group had increased Path ratio (mean difference = 0.04, *P* ≤ 0.001, *d* = 1.07), increased Variable error (mean difference = 6 mm, *P* ≤ 0.001, *d* = 3.38), and increased Acromion displacement (mean difference = 11 mm, *P* ≤ 0.001, *d* = 1.61) compared to the control group. Both the stroke group and the control group spent less time to perform return movements (0.61 ± 0.18 s and 0.48 ± 0.10 s, respectively) than to perform pointing movements (cf. Table 2), and like for the pointing movements the persons post-stroke demonstrated less smooth return movements than the controls (NMU 1.9 ± 1.0 and 1.0 ± 0.1, respectively).

**Table 2:** Kinematic outcomes of the Finger-to-Nose test in all participants^a^

Temporal variables	Control group (*n* = 41)	Whole group (*n* = 33)	Stroke group Mild stroke (*n* = 23)	Moderate stroke (*n* = 10)
Total movement time, s	6.80 (1.94)	**9.38 (3.58)** ^**b**^	8.27 (2.51)	11.92 (4.47)^c^
Pointing time, s	0.53 (0.13)	**0.73 (0.24)** ^**b**^	0.64 (0.17)	**0.95 (0.26)** ^**c**^
Peak speed, mm/s	2628 (503)	**2073 (556)** ^**b**^	2265 (524)	1629 (342)^c^
Time to Peak speed, s	0.22 (0.05)	**0.27 (0.06)** ^**b**^	0.25 (0.07)	0.30 (0.05)^c^
Time to Peak speed, %	42.3 (5.3)	38.0 (7.0)^b^	40.2 (5.6)	32.8 (7.4)^c^
Number of movement units, *n*	1.0 (0.1)	1.5 (0.8)^b^	1.2 (0.4)	2.1 (1.2)^c^
IJC shoulder flexion/elbow flexion	0.93 (0.04)	0.96 (0.03)^b^	0.95 (0.04)	0.97 (0.02)
Spatial variables				
Path ratio	1.12 (0.03)	**1.15 (0.04)** ^**b**^	1.14 (0.04)	1.17 (0.04)
Total variability, mm	13.7 (5.0)	23.7 (16.7)^b^	20.3 (11.3)	31.3 (24.2)^c^
Variable error, mm	5.8 (1.7)	**11.7 (7.5)** ^**b**^	11.5 (8.2)	12.2 (6.2)
Range of motion, degrees				
Shoulder flexion	32.2 (10.6)	31.4 (11.7)	28.5 (10.8)	37.9 (11.4)
Shoulder abduction	9.0 (5.1)	7.8 (4.4)	7.4 (4.6)	8.8 (3.9)
Elbow flexion	74.8 (9.9)	69.9 (12.1)	74.3 (11.0)	**59.9 (8.3)** ^**c**^
Forearm rotation	61.5 (14.9)	53.2 (16.4)^b^	54.9 (12.8)	49.2 (23.0)
Acromion displacement, mm	−7.9 (6.6)	**−18.9 (15.4)** ^**b**^	−14.3 (8.9)	−29.6 (21.7)^c^
Nose displacement, mm	1.0 (4.9)	-1.2 (6.7)	-0.1 (5.0)	0.3 (2.6)

^a^Results are reported as mean (standard deviation). *IJC* interjoint coordination. Significant difference between affected arm in persons post-stroke and non-dominant arm in controls (^b^ *P* < 0.05), and between affected arms in persons with mild and moderate post-stroke symptoms (^c^ *P* < 0.05). Bold characters indicate the comparisons that are significant also after Bonferroni correction (*P* ≤ 0.001)

**Fig. 3:**
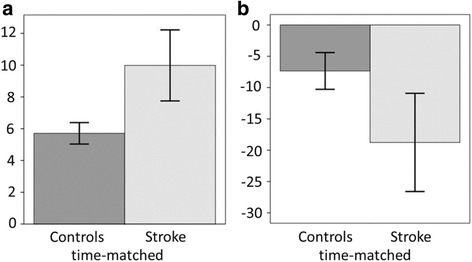
Histograms of variable error and acromion displacement from time-matched subgroup data. Mean and 95% confidence interval for (**a**) variable error (mm) and (**b**) acromion displacement (mm) during the Finger-to-Nose test. Data from 22 controls (*dark bars*) are compared with data from 22 persons post-stroke (*light bars*)

In the analysis of the two time-matched subgroups (22 controls, 22 stroke), consistent differences were found in Variable error (*P* ≤ 0.001, *r* = 0.59), and Acromion displacement (*P* ≤ 0.001, *r* = 0.51), as shown in Fig. 3.

### Construct validity

**Fig. 4:**
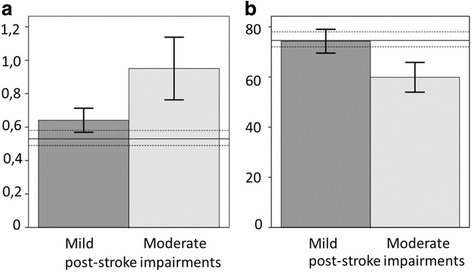
Histograms of pointing time and elbow flexion from stroke subgroup data. Mean and 95% confidence interval) for (**a**) pointing time (s) and (**b**) elbow flexion (deg) during the knee to nose movement of the Finger-to-Nose-Test. Data from 23 persons post-stroke with mild impairments (*dark bars*) and ten persons post-stroke with moderate impairments (*light bars*) are compared with data from the control group (*horizontal lines* indicate mean and 95% confidence interval)

Persons with moderate stroke had significantly slower Pointing time (mean difference 0.3 s; *P* ≤ 0.001; *r* = 0.56) compared to persons with mild stroke (Table 2) and decreased ROM in elbow flexion (mean difference = 14.4°, *P* ≤ 0.001; *r* = 0.56) compared to the persons with mild stroke (Table 2 and Fig. 4).

For the stroke group, TMT was correlated with the NMU, TPS %, Total variability and Path ratio, as shown in Table 3. The Path ratio had the lowest strength of correlation and was not entered in the regression model. The NMU alone explained 60% of the variance in TMT (Table 4). The backward multiple regression revealed that the kinematic variables NMU, TPS% and Total variability together explained 72% of the variance in TMT, demonstrating a unique contribution to the equation (11.5, 9.4 and 3.4%, respectively).

**Table 3:** Correlations between kinematic variables of the Finger-to-Nose Test (33 persons post-stroke)

	TMT	TPS%	NMU	IJC	PR	TV	ROM
Total movement time (TMT)							
Time to peak speed (TPS%)	−0.559**						
Number of movement units (NMU)	0.709**	−0.386*					
Interjoint coordination (IJC)	0.131	−0.327	0.281				
Path ratio (PR)	0.375*	−0.221	0.565**	0.438*			
Total variability (TV)	0.467**	−0.150	0.508**	0.149	0.378*		
Range of motion (ROM) elbow flex	−0.159	0.322	−0.351*	−0.435*	0.064	−0.279	
Acromion displacement	0.151	0.134	−0.280	−0.372*	−0.131	0.111	0.401*

Spearman rank order correlation (* = <0.05, ** = <0.01)

**Table 4:** Multiple Regression Analysis for the Finger-to-Nose Test (33 persons post-stroke)

	Univariate Regression			Extracted Predictors in Backward Regression				
	Unstand B	SE	Adjusted *R* ^*2*^	Unstand B	SE	Part	Adjusted *R* ^*2*^	Model *P*-value
TMT: dependent variable								
Independent variables:							0.718	<.001
NMU	3.364***	0.486	0.595	2.014**	0.559	11.5%		
TPS%	−0.328***	0.070	0.395	−0.177**	0.054	9.4%		
Total variability	0.141***	0.029	0.411	0.052	0.026	3.4%		

*TMT* Total movement time, *NMU* Number of movement units, *TPS%* Time to peak speed in percentage, *Unstand B* unstandardized beta value, *SE* standard error, *Part* partial correlation coefficient. (** = <0.01, *** = <0.001)

## Discussion

To our knowledge, this is the first study to investigate the construct validity of the FNT, as performed in the FMA-UE, based on kinematic analyses. The results indicated that four temporal and three spatial of 16 investigated kinematic variables during performance of the FNT differed between controls and persons post-stroke. The TMT to perform the FNT was prolonged for the stroke group, which is consistent with prior studies of pointing tasks in persons post-stroke [19, 22]. Nevertheless, two-thirds of the stroke group had similar movement times as half of the controls. This may be explained by the predominantly mild impairments in the present stroke group. The stroke group had also less straight and less accurate movements, indicated by increased Path ratio and end-point errors, in agreement with prior studies of pointing movements [19, 20, 22]. Previous studies have reported high variability in accuracy during forward reaching post-stroke [20, 30, 34]. One of these studies compared two reaching conditions and found slightly higher end-point errors in reaching upwards to a target compared to reaching forward to a target [30]. Indeed, our stroke group also demonstrated high variability in end-point errors when pointing upwards to the nose. Although both groups in our study demonstrated similar variability in Path ratio and ROM in the shoulder and elbow joints, the stroke group showed higher variability in end-point errors compared to the control group. Interestingly, even though a subgroup of persons post-stroke had equal movement time as the controls, they still demonstrated reduced accuracy and compensatory movements. Thus, the 3D motion analysis captured deviations in pointing movements post-stroke which were not directly time-dependent.

The interjoint correlation values were high between shoulder and elbow joints of both the control group and the stroke group, but especially in the affected arm (IJC close to 1), representing a high coupling between these joints. In analogy with our findings, a study examining upward reaching towards a target close to the head (requiring shoulder and elbow flexion as in the FNT), found that persons post-stroke tended to produce concurrent flexion of both elbow and shoulder joints [30]. In contrast to our results, another study showed lower cross-correlation between shoulder and elbow joints post-stroke [23]. In that study, however, pointing towards an external target (requiring shoulder flexion and elbow extension) was investigated. Hence, a disrupted interjoint coordination during pointing movements post-stroke may be either an abnormally higher or lower coupling between shoulder and elbow joints depending on the task condition. Reaching upwards to a target is usually easier to perform compared to forward reaching post-stroke, which is probably related to the task requiring a sustained flexor synergy and a shorter lever arm [30]. Sensorimotor control of egocentric movements (towards the body), as in the FNT, may differ from that of exocentric movements (away from the body) towards an object in extra-personal space. In both eyes-closed cases, the brain has to rely on a body-centered coordinate system instead of an eye-centered coordinate system to locate the target [35]. However, the sensory feedback in the egocentric reaching task is obtained by proprioceptive information from the upper limb joints including mutual tactile information from the touch creating a sensory mapping that is based on signals from both the fingertip and the nose tip. The source of feedback is hence intrinsic, i.e. arises from the individual’s own sensory systems [36].

A recent review concluded that trunk restraint is a beneficial method to limit compensatory movements during reaching post-stroke especially for those with moderate-to-severe impairments [37]. In this study, however, there were no restrictions regarding head and trunk movements since this is not done in usual clinical practice. The stroke group used excessive scapular and trunk motions during the Pointing phase, as revealed by increased displacement of the acromion marker, while their head motion was similar to head motion of control subjects, as shown by equal displacement of the nose marker. During a forward reaching task, persons post-stroke have demonstrated compensatory *anterior* sagittal movements such as trunk forward bending [38] or scapular protraction [37]. In our study of a point-to-nose task, the stroke group demonstrated a compensatory *posterior* sagittal movement. Hence, excessive trunk/scapular movements during pointing or reaching may occur in either direction in stroke patients depending on the task. During forward reaching, it has been shown that trunk sagittal displacement occurs simultaneously with decreased shoulder flexion and decreased elbow extension post-stroke [20]. In our study, however, there were few significant differences between the stroke group and the control group regarding ROM of shoulder and elbow joints. Unexpectedly, the stroke group did not display increased shoulder abduction during the FNT which is in contrast to results of earlier studies that have investigated hand-to mouth tasks in persons post-stroke [12, 25]. Within the stroke group, persons with moderate impairments had more marked kinematic deviations from controls compared to persons with mild impairments. Prolonged Pointing time and decreased ROM in elbow flexion during the pointing phase were particularly pronounced for those who had moderate impairments. The latter may have been caused by a more flexed elbow at start position and/or use of excessive scapular and trunk movements during the pointing phase that resulted in less elbow flexion. However, as the mild and moderate impairments were unequally distributed, this should be interpreted with caution.

No variables of the Return phase were entered in the regression model since we focused on the egocentric part of the FNT. Within the stroke group, TMT was highly correlated with smoothness (NMU of the Pointing phase *r*_*s*_ = 0.71), a feature that is assumed for well-coordinated movement [17, 18]. A high correlation between TMT and NMU has also been reported in persons post-stroke during a drinking task, where it was suggested that the TMT could be used as an indirect measure of movement smoothness [12]. However, a prior study concluded that slower movement speed does not entirely explain the increased temporal segmentation of endpoint movement evident in persons with stroke [20]. Our stroke group showed a moderate correlation between TPS% and TMT (*r*_*s*_ = 0.56). The persons post-stroke had lower amplitudes of Peak speed, prolonged deceleration phases, and left-shifted velocity profiles when moving their finger towards the nose compared to controls. The velocity profiles in our study groups are comparable to those velocity profiles seen in similar groups during a glass-to-mouth task [12], where controls had only one movement unit while persons post-stroke had multiple movement units and lower peak speeds. Other indirect measures of coordination are movement energy efficiency and accuracy [39], which were represented in this study by Path ratio and Total variability. Those spatial variables were, however, only weakly correlated with TMT. Notably, TMT did not correlate with IJC, which may seem contradictory for a coordination test such as the FNT.

The strongest associations, according to the multiple regression model, were found between the time to perform the FNT (TMT) and NMU, TPS% and Total variability. Total variability alone was weakly associated with TMT. This indicated that faster performance of FNT in a person post-stroke did not necessarily reflect more precise finger-to-nose contact. It may be that movement accuracy was sacrificed for increased speed, in which case a less accurate movement might risk being misinterpreted as an improvement of coordination [40].

Although the movement time measured by a stopwatch may be considered as an easy and ‘objective’ measure of the FNT, there are limitations. First, a person with mild stroke may have altered upper limb movements albeit the movement time is comparable with healthy subjects. Second, a reduction in time to perform the FNT between two evaluations may be a ‘false’ improvement as accuracy may be sacrificed in favor of faster speed. As the instructions of the FNT encompass two difficulties; 1) touch nose without vison and 2) as fast as possible, this dual task command may lead to different movement times depending on task priority (cf. Fitts’ law [41]). Third, the time-monitored FNT corresponds to movement speed and is mainly associated with movement smoothness (temporal end-point variables) but not to joint motions and compensatory movements (spatial variables). For a coordination test, both temporal and spatial aspects are of importance. Fortunately, more recently developed scales aim to assess quality of movement during goal-directed tasks in persons with stroke [42–44]. Such scales are good alternatives or supplements when assessing multi-joint coordination in the stroke-affected arm.

### Limitations

The results of this study cannot be extended to a stroke population with severe impairments. Although our stroke sample does not fully represent the broad range of post-stroke hemiparesis, they are an important subpopulation as they may suffer from subtle deficits that are not clearly identified. Additionally, the sample size restricted the number of variables entered in the regression model and the power of the sub-group analysis. At the same time, the sample size (>30 in each group) is considered relatively large for a kinematic study of goal-oriented arm movements in persons post-stroke [26]. This study was not designed to compare data between stroke groups with different severity, instead we aimed to investigate relationships between movement assessment variables and severity of stroke symptoms. The results indicate that almost half of the selected kinematic variables are indeed sensitive to stroke severity, and we suggest that this should be evaluated in a larger stroke population. Because coordination of reaching is complex, multiple variables were included in the analysis to represent different movement characteristics. To counteract the problem of multiple comparisons, a Bonferroni correction was employed. Although this correction may be a simple and effective way of avoiding Type I errors (detecting a difference that is not truly present), it is very conservative and therefore strongly increases the risk of Type II errors (failing to detect a difference that is present) [45]. Finally, the results of this study are based on kinematic outcomes of the pointing phase and do not take into account the movement performance throughout the whole test.

## Conclusions

The timed FNT discriminates between persons with mild and moderate upper limb impairments. However, the time to perform the FNT should not be considered as an estimate of upper limb coordination in persons post-stroke as it does not sufficiently reflect spatial aspects of upper limb coordination and possible use of compensation. The crude ordinal rating of dysmetria and tremor, that is included in the FMA-UE, also fails to consider compensatory movements. Therefore, clinical scales that capture qualitative aspects of upper limb and trunk movements should be included when assessing upper limb coordination after stroke. Kinematic analysis certainly provides an added value through a more comprehensive and valid evaluation of upper limb movement post-stroke, that detects differences in pointing movements that are not captured in the timed FNT.

## Abbreviations

FMA-UE: Fugl-Meyer assessment scale for the upper extremity
FNT: Finger-to-nose test
IJC: Interjoint coordination
NMU: Number of movement units
ROM: Range of motion
TMT: Total movement time
TPS: Time to peak speed
